# Provider survey to assess the usability and acceptability of an automated electronic health record-based tool for atrial fibrillation to improve anticoagulation management

**DOI:** 10.1093/jamiaopen/ooag055

**Published:** 2026-04-18

**Authors:** Brittany A Carlson, Catherine P Benziger, Melissa L Harry, Nicole A Groth, Clayton I Allen, Laura A Freitag

**Affiliations:** School of Medicine, University of Minnesota Medical School-Duluth Campus, Duluth, MN, United States; Heart and Vascular Center, Essentia Health, Duluth, MN, United States; Essentia Institute of Rural Health, Essentia Health, Duluth, MN, United States; Essentia Institute of Rural Health, Essentia Health, Duluth, MN, United States; Essentia Institute of Rural Health, Essentia Health, Duluth, MN, United States; Essentia Institute of Rural Health, Essentia Health, Duluth, MN, United States

**Keywords:** atrial fibrillation, atrial flutter, anticoagulation, clinical decision support systems, primary healthcare care gap, electronic health records

## Abstract

**Objective:**

Implemented a tool to identify high-risk patients with atrial fibrillation (AF) with a CHA_2_DS_2_-VASc score of ≥2 (males) or ≥3 (females) who are not treated with oral anticoagulation. Aimed to evaluate the acceptability and usability of the “AF or Flutter not on Anticoagulant” electronic health record-based Care Gap (AF Care Gap) alert and associated best practice advisory (BPA) for clinicians managing patients with AF.

**Materials and Methods:**

An electronic survey was sent to 490 primary care and cardiology providers at Essentia Health (Duluth, MN, USA) to evaluate the usability, acceptability, and obtain feedback post-implementation. We excluded providers who did not complete the consent (*n = *9), give consent (*n = *15), complete the survey (*n = *340), or see AF patients (*n = *5).

**Results and Discussion:**

Survey response rate was 25% (*N = *121); 51% reported prior use of the AF Care Gap (*N = *62) with the majority (73%) in family medicine. Most users and nonusers reported they were “likely/extremely likely” to start a conversation about anticoagulation and use the AF Care Gap or BPA in their future practice (84%). Of those who used it, 75% of providers were “likely/extremely likely” to prescribe anticoagulation. Most users would recommend it to others (67%). On the System Usability Scale, the AF Care Gap scored 72.5/100. The acceptance was 27/35 using a modified Theoretical Framework of Acceptability questionnaire.

**Conclusion:**

Survey respondents report above average usability and acceptability. Future evaluation of the AF Care Gap tool utilization and persistent gaps in anticoagulation management are still needed to improve management for high-risk AF patients.

## Background and significance

Atrial Fibrillation (AF), the most common arrhythmia, is a global epidemic with a near linear prevalence projection paralleled with age and is projected to be 62 million cases by 2050.^1–3^ Patients with AF are at five times higher risk of developing thromboembolism which leads to stroke, the second leading cause of disability and death.^4^^,^^5^ AF is the cause of 15–20% of all strokes in the United States.^2^ Substantial research has found oral anticoagulants (OACs) are highly effective at reducing the risk of ischemic stroke.^6^ Despite the prevalence and risk of AF and highly effective OAC therapy, studies show only half of high-risk AF patients are being treated and of those treated less than 70% are receiving the adequate dose.^2^^,^^6^^,^^7^ The rapidly increasing prevalence of AF due to an aging population and limited access to subspecialists, particularly in rural areas, has forced primary care providers to initiate and manage AF OAC.^8^^,^^9^ With this new task may come with fears of correct dosing.^10^

To assist primary care providers in OAC prescription, an automated risk calculator, the CHA_2_DS_2_-VASc calculator,^11^ was built into the electronic health record (EHR) (Epic Systems, Verona, WI). The risk calculator works to estimate the risk for thromboembolism and to identify high-risk patients. Per American College of Cardiology/American Heart Association/Heart Rhythm Society (AHA/ACC/HRS) guidelines, high risk patients with AF or atrial flutter include males with a CHA_2_DS_2_-VASc (Congestive Heart Failure/Left Ventricular Dysfunction, Hypertension, Age≥ 75, Diabetes mellitus, Stroke/Transient Ischemic Attack/Thromboembolism, Vascular disease, Age 65-75, Sex category (female gender)) score of 2 or higher and females with a score of 3 or higher that do not have moderate or severe mitral stenosis or mechanical heart valves and recommend OAC therapy.^12^ Bleeding risk is a common side effect of OAC. The HASBLED (Hypertension, Abnormal Liver or renal failure, Stroke, Bleeding, Labile INR, Elderly [age >65], Drugs or Alcohol) and Anticoagulation and Risk Factors in Atrial Fibrillation (ATRIA) have bleeding risk scores developed to help evaluate risk of bleeding for patients with AF.^13^ Similar to the CHA_2_DS_2_-VASc calculator, the ATRIA bleed risk score used variables that can be automatically calculated and displayed in the EHR. Despite implementation of the automatic risk calculators, in 2018, previous research at Essentia Health (EH) found one in four high risk patients with AF or flutter were not receiving OAC therapy.^8^ Several studies have investigated barriers adding to the lack of prescription OAC of high-risk AF and include both patient and provider hesitancies. Examples include providers’ comfort level with knowledge and understanding of oral anticoagulants, years of training and lack of confidence in identifying high-risk AF.^2^^,^^14^

In lieu of increasing numbers, the EH team developed an automated EHR-based clinical decision support tool, which was sent to providers who work with patients that have AF. The survey helps identify Care Gap alerts from the health maintenance section of the EHR called, “AF or Flutter not on Anticoagulant” (AF Care Gap). Clinical decision support tools have been implemented as tools to assist providers in improving patient care and reducing costs.^15^ Additional artificial intelligence (AI)-based models are also showing promising potential but with limited evidence on clinical outcomes.^16–18^ The AF Care Gap is a clinical decision support tool that utilizes EHR data to identify patients by ICD-10 diagnostic codes with the diagnosis of AF or atrial flutter, who are not currently on anticoagulation medication and meet high risk criteria for a thromboembolic stroke based on the CHA_2_DS_2_-VASc score (≥ 2 males; ≥ 3 females) and recommends initiation of OAC. From the AF Care Gap, providers are given the option to open an Epic SmartSet (order set) to order anticoagulation therapy, baseline labs, and send a referral to anticoagulation clinic, as needed (Appendix A, available as supplementary data at [*JAMIA Open*] online). In total, there are six ways to satisfy the AF Care Gap: (1) prescription of OACs; (2) documentation of contraindication to anticoagulation; (3) referral and placement of WATCHMAN^TM^ left atrial appendage occlusion device; (4) deferral of alert to a later date; (5) removal of the diagnosis from problem list if in error; (6) discontinuation of the AF Care Gap due to end of life, age of patient, severe dementia, on hospice or other patient or provider request. The goal of this tool is to guide primary care providers to prescribe OACs for high-risk AF patients to reduce stroke.

## Objective

We aimed to evaluate the acceptability and usability of the AF Care Gap alert and associated best practice advisory (BPA) for patients with AF in a cross-sectional survey of primary care and cardiology. Evaluation of a new tool like the AF Care Gap is important to help improve acceptance and use of the tool to help narrow this gap in care for AF patients and reduce risk of stroke and its complications.

## Materials and methods

Beginning in March 1, 2023, we implemented an automated EHR-based tool for qualifying patients with AF or atrial flutter as identified by ICD-10 diagnostic codes. At the time of the survey reported in this study, the AF Care Gap had been available for nine months. Providers were offered one opportunity for a training session, which was before the AF Care Gap was released. Various screening tests that are part of the health system quality metrics and annual health maintenance visits have moved onto the Epic health maintenance section and have associated Care Gap alerts rather than BPA alerts. We focused our assessment on the AF Care Gap alert primarily as the AF BPA alert is not a pop up but lives in the BPA alert section in Epic.

**Figure 1.**
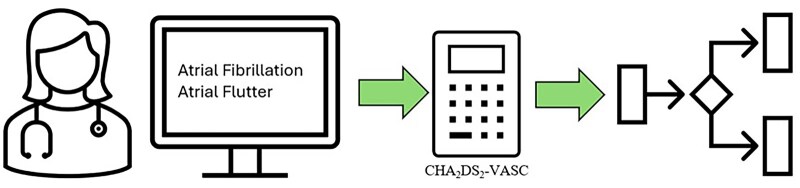
Simplified clinical decision flow chart. At a clinic encounter, a patient with atrial fibrillation or atrial flutter on the problem list triggers automatic calculation of the CHA2DS2-VASc risk score. Based on the score and the medication list, the tool would determine if the patient with atrial fibrillation or atrial flutter was or was not meeting guideline recommendations for anticoagulation. An Epic CareGap and Best Practice Advisory alert and SmartSet would be shown if they do not meet current guidelines. CHA_2_DS_2_-VASc, Congestive Heart Failure/Left Ventricular Dysfunction, Hypertension, Age≥ 75, Diabetes mellitus, Stroke/Transient Ischemic Attack/Thromboembolism, Vascular disease, Age 65–75, Sex category (female gender).

The AF Care Gap alert work flow is as follows: an automated electronic CHA_2_DS_2_-VASc calculator is triggered during clinic visits for qualifying patients and displayed to providers.^19^ Based on this information, a clinical decision support system including an Epic Care Gap (recommended services) activity and BPA alert is triggered to alert providers if there is a patient at high risk for stroke or thromboembolism who is not on current anticoagulation (Figure 1).

Other healthcare systems have implemented the CHA_2_DS_2_-VASc calculator into the EHR using clinician decision support.^20^^,^^21^ However, few papers have been published describing tools similar to the AF Care Gap. One Swedish study described a tool, which alerts clinicians as a general pop-up warning when a patient has AF added to their problem list, a CHA_2_DS_2_-VASc risk factor and is not currently on anticoagulation.^22^ The differences in the AF Care Gap include that it is not a general pop up as it displays in the health maintenance section. The algorithm in the background will also calculate the CHA_2_DS_2_-VASc score prior to display and it only displays if patient meets high-risk criteria. Lastly, it is connected to an order set to prescribe anticoagulation, send referrals and order follow up lab work.

As of November 14, 2025, the Care Gap tool and BPA alert had identified 7653 patients with atrial fibrillation or flutter not on anticoagulation. The use of the AF BPA alert was 6.3% (*N = *482) with providers having clicked “acknowledged or override” alert the majority of the time (*N = *338), followed by opening the anticoagulation order set (*N = *131) or adding an exclusion problem to problem list (*N = *100). Providers interacted with the Care Gap tool less often either discontinuing (*N = *227) or postponing (*N = *11) it with a majority of patients (*N = *3,002) still with overdue status. However, if anticoagulation was ordered then the Care Gap tool would be satisfied even if providers did not directly interact with it. The outcome of the AF Care Gap and BPA alert tools on anticoagulation management in patients with a CHA_2_DS_2_-VASc score of ≥2 (male) or ≥3 (females) who were not being treated with oral anticoagulation are described in Appendix A, available as supplementary data at [*JAMIA Open*] online and shown in Figure 2 in Brod et al. and outcomes are still being analyzed.^19^

**Figure 2.**
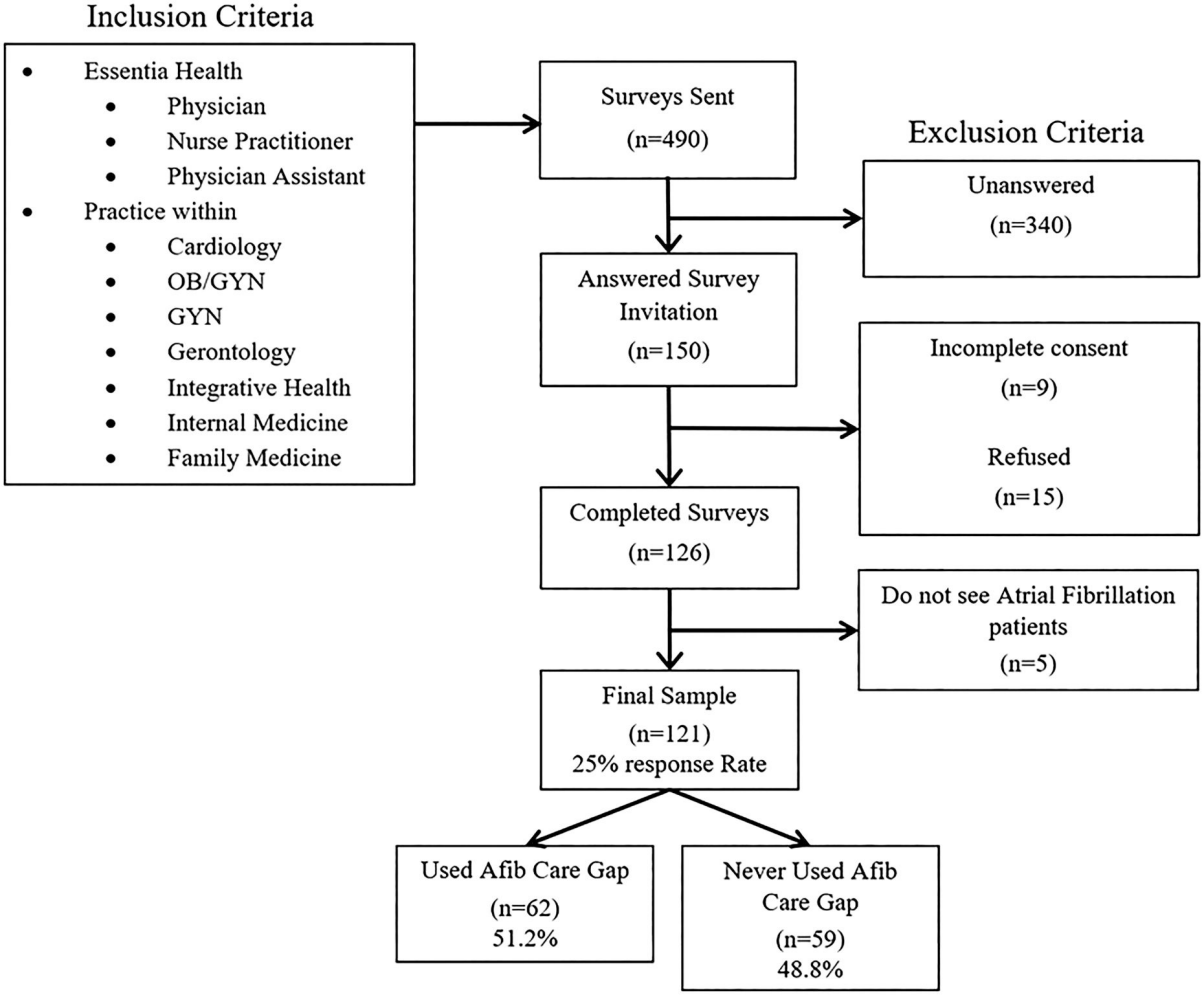
Flowchart for respondents in the Epic Atrial Fibrillation Care Gap provider survey. GYN, gynecology; OB/GYN, obstetrics/gynecology.

### Study population

We conducted a post-implementation web-based cross-sectional survey of Essentia Health primary care and cardiology physicians and advanced practice providers to assess the usability and acceptability of the AF Care Gap. Between January 15, 2024 and February 29, 2024, a total of 490 providers were sent emails to complete the survey. Providers included family medicine, cardiology, internal medicine, gerontology, and integrative health specialties across 3 Midwest states (Minnesota, Wisconsin, North Dakota).

### Study design

The survey was distributed using REDCap electronic data capture tools,^23^^,^^24^ which included sending an initial email invitation and four email reminders to participants. The survey was administered over a 6-week period and was voluntary. No compensation was rewarded for provider participation. Respondents completed the informed consent form electronically. The survey included 25 questions related to the acceptability, usability, and general feedback of the AF Care Gap and BPA. Most questions were multiple choice with five answers available using the Likert scale, with other select all that apply questions and a few were short answers.

We utilized the System Usability Scale (SUS) within our survey to evaluate the usability of the AF Care Gap tool in those respondents who reported they had used the tool.^25^ The SUS provides the overall score of system usability ranging from 0 to 100.^25^ It consists of 10 questions on a 5-point Likert scale ranging from 1—strongly disagree to 5—strongly agree.^25^ Scoring involves subtracting 1 from all odd items and subtracting all even items from 5, which scales each item from 0 to 4.^25^ The total is then multiplied by 2.5 to provide the overall SUS score.^25^

We also utilized the Theoretical Framework of Acceptability to help assess seven constructs of acceptability: affective attitude, ethicality, burden, perceived effectiveness, intervention coherence, opportunity cost and self-efficacy.^26^^,^^27^ In the context of this study, we modified the Theoretical Framework of Acceptability to exclude perceived effectiveness in order to evaluate the overall acceptability and correlate future use and future recommendation of the AF Care Gap with acceptability (survey questions shown Appendix B, available as supplementary data at [*JAMIA Open*] online). The Theoretical Framework of Acceptability has been used in studying the acceptability of other clinical decision support tools, digital tools, and EHR-based processes.^28^^,^^29^

The flow diagram used to determine our final sample is shown in Figure 2. After the collection of the survey results, unanswered surveys and incomplete surveys were removed. Of the completed surveys, any incomplete consent or those who did not consent were removed. Finally, respondents who reported not managing AF patients were removed. Data was collected within REDCap and exported in an Excel format for statistical analysis. First and last names were collected for consent purposes only. All questions were anonymized before analysis. The study protocol was approved by the study health system’s Institutional Review Board (EIRH-23-1984).

### Statistical analysis

Survey responses were analyzed using Excel and International Business Machines’ (IBM) Statistical Package for the Social Sciences (SPSS) version 29. Descriptive statistics were used to summarize physician demographics and responses to usability, acceptability, and general feedback of the AF Care Gap. For continuous baseline variables, we reported means and standard deviation or medians with 25th and 75th percentile interquartile ranges and made comparisons using student’s *t*-test or ANOVA if appropriate. For categorical variables, we reported frequencies and percentages with results from the chi-squared test. Following descriptive analysis, Pearson’s correlations were used to identify relationships between aspects of acceptability and future use and future recommendation. A *P*-value of <0.05 was considered statistically significant.

## Results

### Demographics

A total of 121 surveys were included in this analysis (25.0% response rate) of 490 sent (Figure 2). Demographics of respondents stratified by those that reported using the AF Care Gap compared to those who reported never using the AF Care Gap are shown in Table 1. Family medicine (59.5%) and cardiology (25.6%) were the most reported specialties with even distribution of physicians (51.2%) and advanced practice providers (nurse practitioner [NP] and physician assistant [PA]) (48.8%). At the time of the survey, a majority had been practicing for 1–5 years (38.0%) within primarily clinic-based practices (66.9%) and nearly half (43.7%) were in rural/small town areas (<10 000 population). A majority (52.1%) reported having seen over 50 patients with AF in the past 12 months.

**Table 1.** Baseline demographics in the atrial fibrillation care gap provider survey.

Demographic profile	Final sample	%	Used Afib care gap	%	Never used Afib care gap	%
***N* = (unless specified)**	121		62	51.24	59	48.76
***Professional Role***						
**Physician (MD, DO, MBBS)**	62	51.24	37	59.68	25	42.37
**Advanced practitioner (NP, PA**)	59	48.76	25	40.32	34	57.63
***Medical Specialty***						
**Family medicine**	72	59.5	45	72.58	27	45.76
**Internal Medicine**	12	9.92	5	8.06	7	11.86
**Cardiology**	31	25.62	8	12.9	23	38.98
**Other (Integrative Health, Gerontology)**	6	4.96	4	6.45	2	3.39
***Years of Practice***						
**1 Year**	10	8.26	6	9.68	4	6.78
**1–5 Years**	46	38.02	22	35.48	24	40.68
**6–10 Years**	22	18.18	13	20.97	9	15.25
**11–15 Years**	18	14.88	8	12.9	10	16.95
**16–20 Years**	5	4.13	1	1.61	4	6.78
**>20 Years**	20	16.53	12	19.35	8	13.56
***Primary Location***						
**Rural/Small town** **(<10,000 population)**	52	43.7	31	51.67	21	35.59
**Suburban/Urban (>10,000 population)**	67	56.3	29	48.33	38	64.41
***Practice Location***						
**Hospital based**	12	9.92	3	4.84	9	15.25
**Clinic based**	81	66.94	42	67.74	37	62.71
**Mix hospital/clinic**	27	22.31	12	19.35	15	25.42
**Assisted Living Facility**	9	7.44	9	14.52	0	0
**Other**	5	4.13	2	3.23	3	5.08
***Number of Afib Patients Seen in 12 Months***						
**1–9**	10	8.26	5	8.06	5	8.47
**10–19**	23	19.01	12	19.35	11	18.64
**20–49**	25	20.66	17	27.42	8	13.56
**>50**	63	52.07	28	45.16	35	59.32

*Note*. Percentages may not add up to 100% due to rounding.

### Clinical decision-making tools

Of all respondents, 66.7% report using electronic health based (Epic) health maintenance tab routinely. Over half (57.0%) have seen the AF Care Gap and 51.2% have used the AF Care Gap (Appendix A, available as supplementary data at [*JAMIA Open*] online). Of those who have used it, 30.7% used it weekly, 29.0% used it monthly, and 40.3% used it less than monthly. A total of 59 respondents (48.8%) reported never using it.

### Survey results

**Figure 3.**
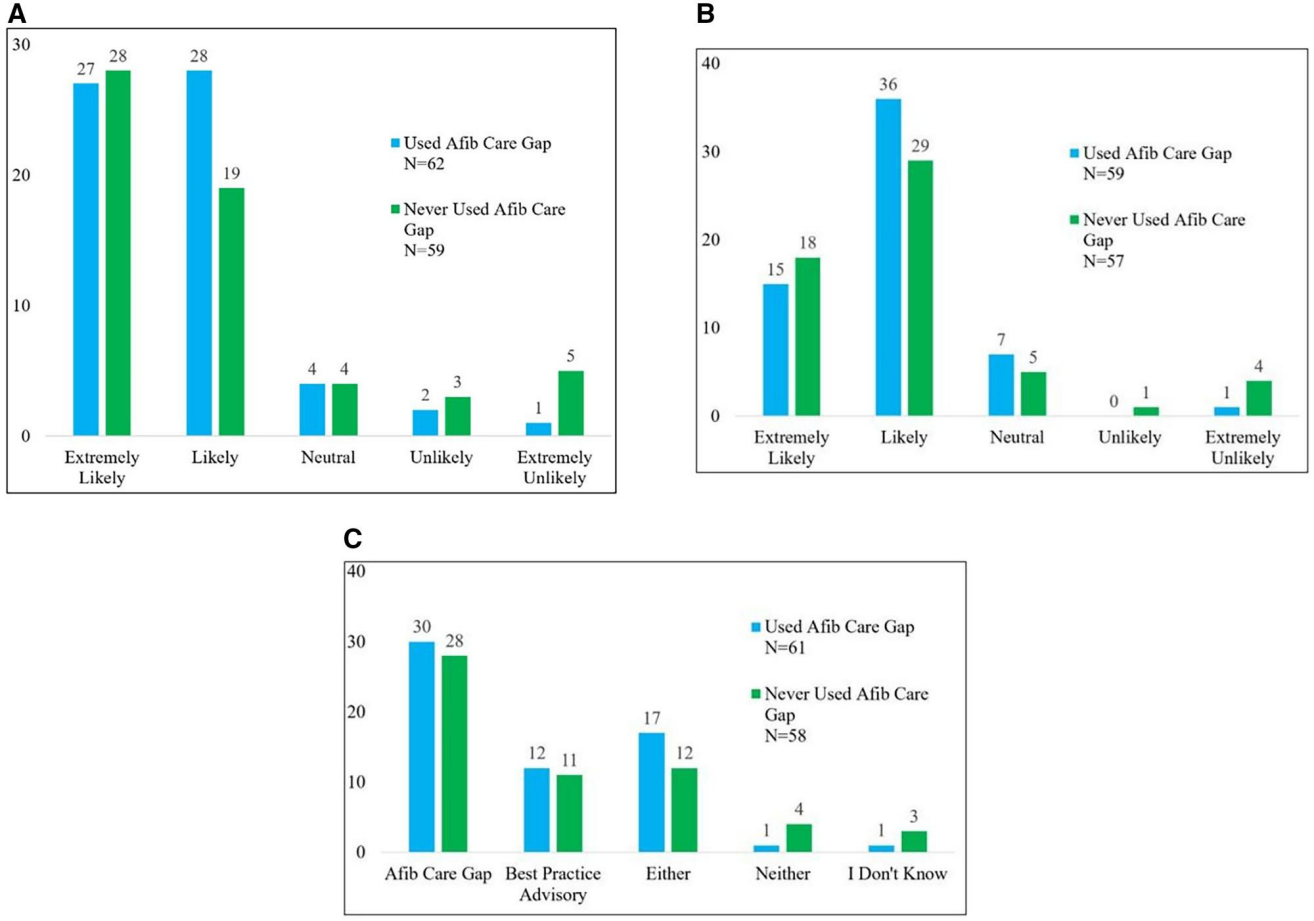
Response to survey questions by those who have used the atrial fibrillation care gap compared to those who have never used atrial fibrillation care gap. Afib, atrial fibrillation. (A) How likely or unlikely are you to have a conversation with a patient with atrial fibrillation or atrial flutter about starting anticoagulation as a result of the 'AFib/Aflutter without Anticoagulant’ Care Gap alert? (B) The 'AFib/Aflutter without Anticoagulant’ Care Gap alert utilizes the CHA_2_DS_2_-VASc calculator.) It is displayed for patients diagnosed with nonvalvular atrial fibrillation or atrial flutter without anticoagulation that are high-risk according to (CHA_2_DS_2_-VASc score of >/=2 male, >/=3 female). In your future practice, how likely are you to respond to the 'AFib/Aflutter without Anticoagulant’ Care Gap alert or 'Best Practice Advisory for patients with Atrial Fibrillation and not on an Anticoagulant’? Note: a response was missing for five participants. (C) Which tool are you more likely to respond to in Epic? Note: a response was missing for two participants.

The main results of the questionnaire are shown in Figure 3. When being alerted by the AF Care Gap, the majority of both those who have used and have never used the AF Care Gap were “extremely likely” or “likely” to have a conversation with their patients about anticoagulation (Figure 3a). Of both those that have used and have never used the AF Care Gap, a majority (81.0%) were “extremely likely” or “likely” to use the AF Care Gap or BPA in their future practice (Figure 3b). Overall, 90.9% of all respondents’ reported preference for AF Care Gap (48.0%), BPA (19.0%), or either (24.0%) (Figure 3c). Of those who have used the AF Care Gap when being alerted (not shown), a majority were “extremely likely” or “likely” to start anticoagulation (75.0%, *n = *47) and 63.0% (*n = *40), were “extremely likely” or “likely” to recommend the AF Care Gap or BPA to other colleagues or clinics. Some other free text suggestions for notifying providers were: “I would prefer to see it documented in the patient’s chart,” “BPAs firing in my experience tend to be cumbersome and contribute to alarm fatigue while also forcing me to take on liability for an issue the patient did not come in for and is not interested in addressing at the time,” and “I chart review for all my patients and utilize the search for atrial fib or atrial flutter, then typically have that conversation with them regarding management plans. An alert within Epic would just be more time consuming because even if it alerted me, I would still do my chart search to confirm diagnosis with EKG/monitor/echo/clinic note etc.”

### Features

Of the care gap alert features, most respondents found the treatment recommendations (75.0%), awareness that patients are not on anticoagulation (73.0%), and anticoagulation dosing information (73.0%) most helpful. Respondents who noted the AF Care Gap order panel was helpful included comments praising the anticoagulation clinic referral. Examples of responses from participants who responded that nothing was helpful about the AF Care Gap alert included: “I personally do not use the Care Gap category,” “I talk about OAC with all my patients with an AF diagnosis,” “I have a good understand of what needs to be done for patients with AF,” “I have not used it yet.”

Like the AF Care Gap, features of the BPA that respondents found most helpful include awareness that the patients are not on anticoagulation (64.0%). However, in the BPA respondents found awareness that the patient has AF or Atrial flutter diagnosis (61.0%) and awareness that the patient meets high-risk criteria (60.0%) also helpful. Some other aspects respondents said were helpful about the BPA were “documentation that risks versus benefits has been completed with patients and they decline anticoagulation,” like the Care Gap and its order panel. Reasons respondents said nothing was helpful about the BPA included “I don’t use the tool,” “outside scope of practice,” and “Again, it is firing for patients with a history of afib/flutter where an anticoagulant is not currently indicated (like post successful ablation). There is no contraindication to anticoagulation per se. It is not indicated now. There is no shared decision making to be done.”

### Usability and acceptability

**Figure 4.**
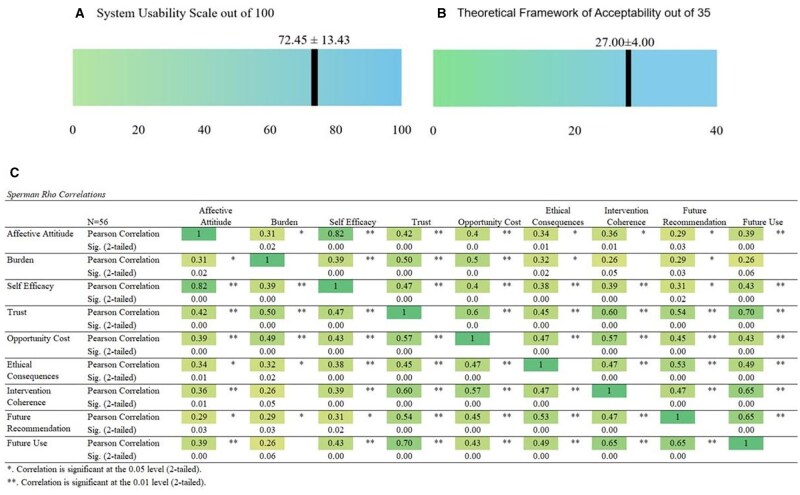
Atrial Fibrillation Care Gap Usability (A) and Acceptability (B) scores with correlation between acceptability and future use and future recommendation (C).

The mean SUS score was 72.5 (*SD* 13.4) (Figure 4a). On the Theoretical Framework of Acceptability questionnaire, the AF Care Gap had an average out of 27 (*SD* 4.0) out of 35 (Figure 4b). Most respondents agreed or strongly agreed they felt comfortable responding to the AF Care Gap (affective attitude = 66.1%) and felt confident responding to the AF Care Gap alert (self-efficacy = 75.0%). Most respondents agreed or strongly agreed that they felt the AF Care Gap alert does not interfere with other priorities while interacting with their patients (opportunity costs = 69.6%) and it was clear to them how the AFib Care Gap alert helps prevent life threatening ischemic stroke for patients with atrial fibrillation or flutter (intervention coherence = 83.9%). However, few respondents felt the AF Care Gap alert requires minimal effort to respond to (burde*n = *3.6%), the AF Care Gap alert does not have negative ethical consequences (ethical consequences = 5.4%), and that they trust the AF Care Gap alert (trust = 1.8%).

Correlations between the aspects of the Theoretical Framework of Acceptability questionnaire, future use and future recommendation are shown in Figure 4c. All correlations were positive, and the majority were statistically significant. Strong correlations were seen between “affective attitude” (comfortable) and “self-efficacy” (confident) (0.82), and between “future use” and “trust” (0.70). The highest moderate correlations were seen between: “future use” and both “future recommendation” (0.65) and “intervention coherence” (0.65); “future recommendation” and “seeing no ethical consequences” (0.65); “intervention coherence” and “trust (0.60); and “opportunity cost” and “intervention coherence” (0.57) and “trust” (0.57).

## Discussion

Family medicine and cardiology providers in a large rural health system reported that they were “likely” or “extremely likely” to start a conversation about anticoagulation and use the AF Care Gap or BPA in their future practice. Of those who reported having used the AF Care Gap or BPA, 75% of providers were “likely” or “extremely likely” to prescribe anticoagulation. A majority of these users would recommend it to others. However, administrative data suggests that use of the AF Care Gap may be inflated, as only 6.3% of patients for the AF Care Gap had a provider respond to it in some way as of November 14, 2025. Alert or alarm fatigue, which has been associated with EHR-based alerts that can include decision aids and risk calculators,^30–33^ may have reduced provider uptake of this tool. Additional or refresher education and training may also be needed to increase provider uptake.

Prior research has demonstrated that one in four patients in a sample of high-risk AF patients were not on anticoagulation.^8^ This new AF Care Gap was designed to target this gap. The questionnaire used in this study, based on the SUS and the Theoretical Framework of Acceptability,^25–27^ provided an easy, real-time measurement of professional acceptance and usability of the AF Care Gap alert. Both the SUS and the Theoretical Framework of Acceptability appear to be useful tools in gauging the acceptability of the AF Care Gap alert and other similar EHR-based alerts. The lowest Theoretical Framework of Acceptability area was “trust,” which could be improved through additional training with supporting evidence and case examples on the AF Care Gap alert and other similar tools. We found that, in general, the providers who responded to the survey were pleased with the AF Care Gap and will continue to use it in their future practice with high usability and acceptability reported. While starting anticoagulation is a shared decision-making choice that starts with a conversation between patients and providers, this survey showed an increase in the likelihood of starting a conversation about anticoagulation with the alert. Also, those who have used it reported an increase in likelihood of starting anticoagulation. Together these actions should help decrease this gap and prevent thromboembolic events. The AF Care Gap was designed to help primary care providers start anticoagulation for high-risk patients, which this survey showed it was used by primary care providers who found its features helpful and who would recommend it to other healthcare systems and colleagues.

Other clinical support tools had SUS scores with a range of 66–80,^34–37^ reflecting C to A-grades.^38^^,^^39^ Our tool scored 72.5, which is considered an above average level (or a B-grade) of usability.^38^ Since we used a modified Theoretical Framework of Acceptability questionnaire, and most other studies did not report an average score, it is not possible to directly compare our clinical decision support tool to others. Future research could examine the generalizability of our modified version of the Theoretical Framework of Acceptability. However, our finding that the majority agreed that the AF Care Gap does not interfere with other priorities while interacting with their patient shows anticoagulation management is a priority in their practice for AF patients. For those who have used it, a majority of respondents felt they understood the AF Care Gap and how it works. While more respondents felt confident that they could respond to the AF Care Gap, fewer respondents felt comfortable responding to the AF Care Gap. While most providers are good at learning as they go, feeling less comfortable responding could indicate a need for more training around the details of how the AF Care Gap works, which the low usage rates shown in our administrative data further supports. The majority not agreeing that the AF Care Gap alert has no ethical consequences with use likely corresponds to previous literature of provider hesitancy from fear of harm from increased bleed risk.^40^ Our finding that the majority of the respondents did not agree that the AF Care Gap alert requires minimal effort compares similarly with concerns about many previous clinical decision making tools taking too much time and being too cumbersome.^37^ The GUIDES checklist provides a guide on developing and revising tools like the AF Care Gap to facilitate use,^41^ which the authors will utilize in optimizing the tool based on the results of the present study.

We performed correlations using ordinal variables to help understand relations among various aspects of acceptability and future use and future recommendations. The very high correlation between intervention coherence and future use supports the need to ensure providers understand how these clinical decision-making tools are there to help them rather than interrupt their day. Also, the very high correlation between seeing no ethical consequences of using the AF Care Gap and future recommendation suggests that tools that align with the personal values of a provider are more likely to be recommended to others.

The AF Care Gap was developed without the use of AI. A recent retrospective observational study compared AI and machine learning detection of AF with cardiologists and emergency medicine physician detection.^42^ AI read emergency department EKGs and generated guide-line consistent anticoagulation recommendations based on scores for CHA_2_DS_2_-VASc and HAS-BLED at rates that were higher than emergency room physicians and were similar to cardiologists.^42^ Future research is still needed that would test this AI tool in clinical practice, as well as in comparison with a tool like the AF Care Gap.

Going forward we need to assess the usage of the AF Care Gap and the impact on patient care. We also need to identify gaps remaining with the usage of this AF Care Gap. If successful, future research should assess the impact in a wider distribution of the AF Care Gap, including urban usage, which also has a cardiologist shortage, where primary care providers are having to start and manage anticoagulation.^43–45^

### Limitations

A strength of this study is that Essentia health is located across northern Minnesota, Wisconsin, and North Dakota, which includes a very heavily rural-based practice. Future research could examine the generalizability of our findings to other rural-serving healthcare systems with similar tools. Limitations include the self-report and observational nature of this study, which was not a cluster randomized control trial. The lack of non-random sampling may have introduced bias, and social desirability bias may have influenced respondents’ answers. Our combining the top two responses for a given item may have masked more granular responses on items where all response options were not presented in our tables or figures. In addition, the SUS was only asked of respondents who reported using the AF Care Gap alert. We also lacked data on patient perspectives. A future mixed methods study should investigate this area. A planned future extension of the present study will include patient outcome data, the findings of which will be published separately. Future research could also develop and test methods for increasing use of the alert in clinical practice, such as ongoing or refresher training and education. In addition, comparing results between primary care and cardiology clinicians was beyond the scope of the present study given journal word limits and is another area for future study.

## Conclusion

Respondents reported above average usability and acceptability of the AF Care Gap with a substantial interest in continuing the AF Care Gap or AF BPA alerts to improve anticoagulation management for high-risk AF patients. Next steps for investigation include evaluating if the EHR-based AF Care Gap has made an impact on patient outcomes, including use of anticoagulation as well as bleeding and stroke rates.

## Supplementary Material

ooag055_Supplementary_Data

## Data Availability

Data collected for this study will be made available to others. Requests should be directed to Catherine Benziger at Catherine.Benziger@EssentiaHealth.org

## Data access, responsibility, and analysis

Catherine Benziger had full access to all the data in the study and takes responsibility for the integrity of the data and accuracy of the data analysis.
