# Supplementary material for: Provider survey to assess the usability and acceptability of an automated electronic health record-based tool for atrial fibrillation to improve anticoagulation management
Source: JAMIA Open. 2026 Apr 18;9(2):ooag055. doi: 10.1093/jamiaopen/ooag055 (PMC13091096; doi:10.1093/jamiaopen/ooag055)

Appendix A: Provider Atrial Fibrillation (AFib) Care Gap and Atrial Fibrillation Not on Anticoagulation Best Practice Advisory (BPA) alert

Examples of the electronic health record (EHR)-based tools for identification of patients with atrial fibrillation or atrial flutter not on anticoagulation are shown in this appendix. Supplemental Figure 1 shows a screenshot of the AFib Care Gap (Epic) alert for “atrial fibrillation or atrial flutter not on anticoagulation.” This is located in the storyboard section in Epic under the patient name and demographic information. It includes the title, date that it is due and any prior dates that it was last addressed. Clicking on the green check mark opens the Epic Anticoagulation SmartSet (Supplemental Figure 2) for selecting direct oral anticoagulation (DOAC) or warfarin. Correct DOAC dosing is based on patient weight. Pre-selected labs and referral to anticoagulation clinic orders are displayed for clinicians. The black ”X” closes the alert. A simultaneous BPA alert (now called OurPractice Advisories), is displayed in yellow (Supplemental Figure 3). Opening the SmartSet displays the full recommendations for anticoagulation and alternative options for the clinician besides ordering anticoagulation medication, such as considering a referral to cardiology to discuss left atrial appendage occlusion device placement (e.g., WATCHMAN) or adding conditions to the problem list that would satisfy the care gap, such as ”contraindication to anticoagulation therapy” or “presence of WATCHMAN left atrial appendage closure device.”

Supplemental Figure 1: Atrial Fibrillation (AFib) Care Gap alert.


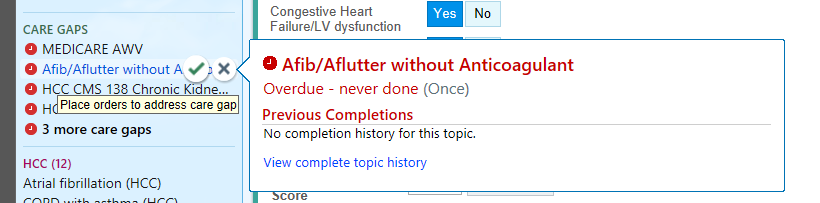


Supplemental Figure 2: Anticoagulation SmartSet with example for selection of apixaban and associated preselected lab and referral to anticoagulation orders.


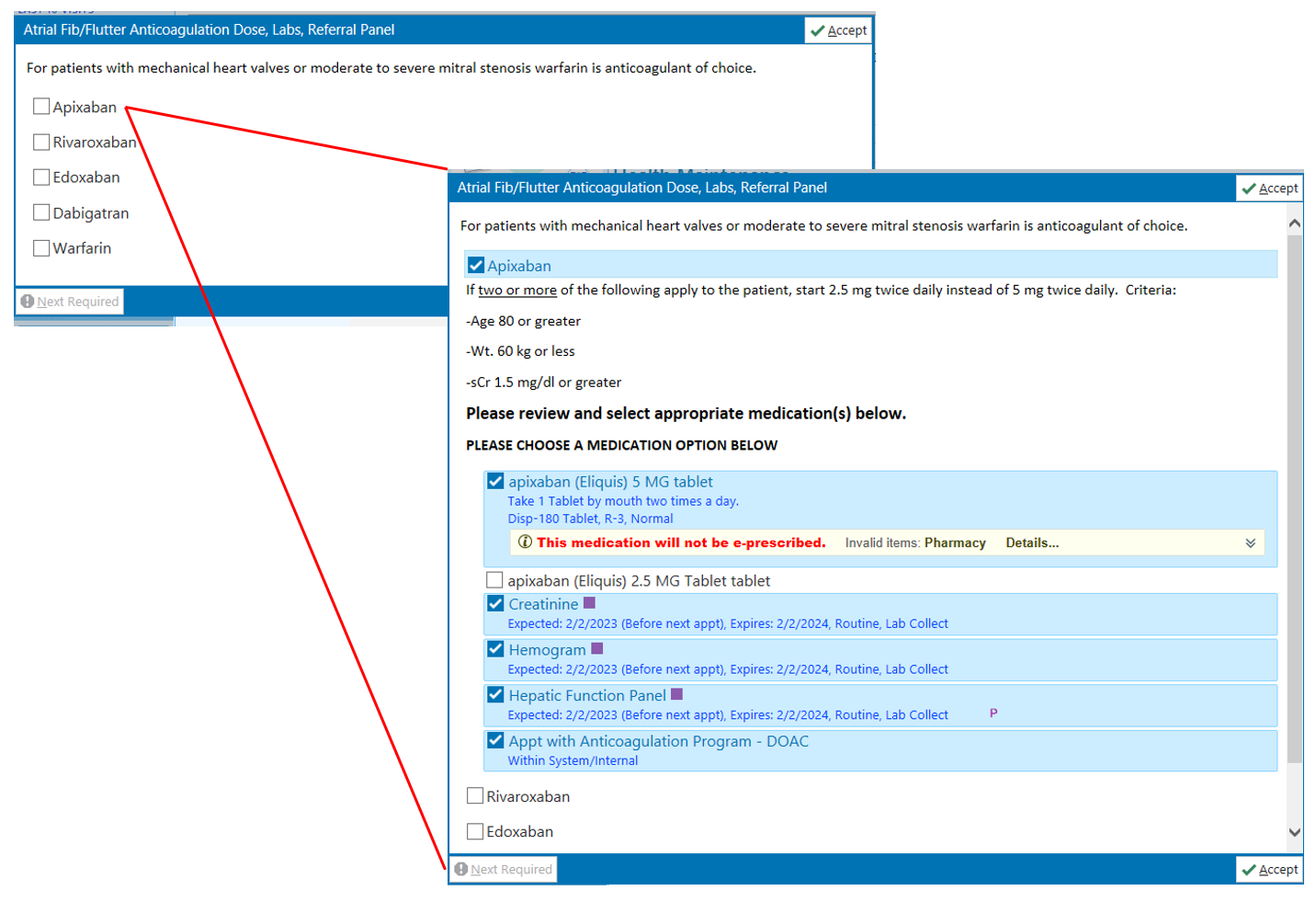


Supplemental Figure 3: Atrial Fibrillation Not on Anticoagulation Best Practice Advisory alert.


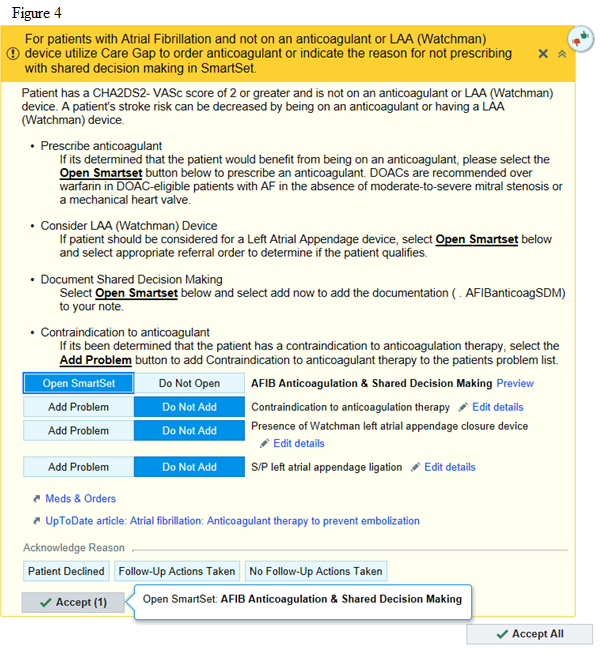

Supplement: ooag055_Supplementary_Data [file ooag055_supplementary_data.zip › Appendix A.docx]
