# Supplementary material for: Provider survey to assess the usability and acceptability of an automated electronic health record-based tool for atrial fibrillation to improve anticoagulation management
Source: JAMIA Open. 2026 Apr 18;9(2):ooag055. doi: 10.1093/jamiaopen/ooag055 (PMC13091096; doi:10.1093/jamiaopen/ooag055)
Supplement: ooag055_Supplementary_Data [file ooag055_supplementary_data.zip › Appendix B_REDCap Data Dictionary-Afib Care Gap Provider Survey.pdf]

Appendix B

Data Dictionary Codebook

13/02/2024 9:07am

#	Variable / Field Name	Field Label *Field Note*	Field Attributes (Field Type, Validation, Choices, Calculations, etc.)
**Instrument: Demographics (demographics)**			
1	[record_id]	Record ID	text<br>Custom alignment: LV<br>Field Annotation: @HIDDEN
2	[first_name]	First Name:	text, Required, Identifier
3	[last_name]	Last Name:	text, Required, Identifier
4	[email]	Email Address:	text (email), Required, Identifier
5	[demographics_complete]	Section Header: *Form Status*<br>Complete?	dropdown<br>0 Incomplete<br>1 Unverified<br>2 Complete
**Instrument: Econsent Form (econsent_form) Enabled as survey**			
6	[consent_form]	Principal Investigator: Katie Benziger, MD, MPH, FAHA, FACCMedical Director, Heart and Vascular ResearchAdjunct Associate Professor, University of Minnesota Medical School, Duluth CampusNon-invasive cardiologist, Essentia Health Heart and Vascular Center407 East Third StreetDuluth, MN 55805Office: 218-786-3443Catherine.Benziger@essentiahealth.org Student Investigator: Brittany Carlson, MS22nd year Medical Student, University of Minnesota, Duluth1035 University Dr.Duluth MN 55812Cell: 320-492-5356Brittany.SkrochCarlson@Essentiahealth.org Consent Information Sheet for Provider Survey Introduction We are conducting research on the newly implemented "Atrial Fibrillation or Flutter not on Anticoagulant" Care Gap (A-Fib Care Gap) at Essentia Health. We are going to give you information and invite you to be part of this research. If you have questions, you can ask any researcher via our contact information below. Purpose of the Research There are many people in the world suffering from non-valvular atrial fibrillation and atrial flutter. Of those, only a portion are receiving treatment. Essentia Health's new A-Fib Care Gap was implemented to help improve the overall treatment of high-risk non-valvular atrial fibrillation and atrial flutter. We want to understand ways to improve this gap. We believe you can help us by telling us	descriptive

about your experience with using the new A-Fib Care Gap. We want to learn how the A-Fib Care Gap tool has been helpful and unhelpful and its acceptability in your community. We also want to know your suggestions for improving the A-Fib Care Gap. Type of Research InterventionThis research will involve your participation in an online survey that will take about 5 minutes. Participant SelectionYou are invited to participate in this research because we feel that your experience as a primary care provider utilizing the Care Gap tool can contribute much to our understanding and knowledge of local health practices. Voluntary Participation Your participation in this research is entirely voluntary. Your choice will have no bearing on your job or any work-related evaluations or reports. Procedures We are asking you to help us learn more about the Atrial Fibrillation Care Gap usage and acceptability in your community. We are inviting you to take part in this research project. If you accept, you will be asked to fill out a survey which will be provided and collected by REDCap, a confidential data collection tool. If you do not wish to answer any of the questions included in the survey, you may skip them and move on to the next question. The survey will be sent via email to all Essentia Health primary care provider staff as a link to the REDCap survey which will collect the information. The information recorded is confidential, your name is not included on the forms, only a number will identify you, and no one else except the research team will have access to your survey. Duration The research takes place over nine months in total. During that time, we will send out the survey which will take about 5 minutes along with four reminders to complete the survey within a five-week period. Risks You do not have to answer any questions or take part in the survey if you feel the question(s) are too personal. Benefits There will be no direct benefit to you, but your participation is likely to help us learn more about the acceptability and usability of the newly released A-Fib Care Gap. Confidentiality We will not be sharing information about you with anyone outside of the research team. The information that we collect from this research project will be kept private. Any information you provide will only be associated with a study ID number. Only the IRB-approved research team will know your number, and this information is kept under lock and key or in a password-protected database with up-to-date firewalls. Sharing the Results Nothing that you tell us will be shared

		with anybody outside the research team and nothing will be attributed to you by name. The knowledge that we get from this research will be shared with the Essentia Health community before it is made widely available to the public. We will publish the results so that other interested people may learn from the research. Right to Refuse or Withdraw You do not have to participate in this research if you do not wish to do so and choosing to participate will not affect your job or job-related evaluations. You may stop participating in the survey at any time that you wish without your job being affected. Whom to Contact for QuestionsIf you have any questions or wish to ask questions later, you may contact the following: Brittany Carlson, MS21035 University Dr.Duluth MN 55812Cell: 320-492-5356Brittany.SkrochCarlson@Essentiahealth.org Katie Benziger, MD, MPH, FAHA, FACC407 East Third StreetDuluth, MN 55805Office: 218-786-3443Page Operator: 218-786-3223Fax: 218-720-4633Catherine.Benziger@essentiahealth.org For questions about your rights as a research participant, please contact the Human Protections Administrator for Essentia Health at 218-786-2540. Bill of Rights for Research Participants As a participant in a research study, you have the right: To have enough time to decide whether or not to be in the research study and to make that decision without any pressure from the people who are conducting the research. To refuse to be in the study at all, and to stop participating at any time after you begin the study. To be told what the study is trying to find out, what will happen to you, and what you will be asked to do if you are in the study. To be told about the reasonably foreseeable risks or discomforts of being in the study. To be told about the possible benefits of being in the study. To be told whether there are any costs associated with being in the study and whether you will be compensated for participating in the study. To be told who will have access to information collected about you, how the information will be used and how your confidentiality will be protected. To be told where to go with questions about the research, about research-related injury, and about your rights as a research subject. If the study involves treatment or therapy: To be told about the other non-research alternative treatment choices you have. To be told where treatment is available should you have a research-related injury, and who will pay for the research-related treatment.	

7	[consent_statement]	Statement of Consent The purpose of this study, the procedures to be followed, the study's risk and benefits have been explained to me. I have been allowed to ask questions, and my questions have been answered to my satisfaction. I have been told whom to contact if I have questions, to talk about problems, concerns or suggestions related to the research, or to obtain information or offer input about the study. I have read this consent form and I agree to be in this study, with the understanding that I may withdraw at anytime. I have been told that I can request a copy of this consent form.As a participant: I have been invited to participate in research about the Atrial Fibrillation Care Gap and local health practices. By participating in this study, I agree to provide the most honest answers I can. I understand any responses I provide will be kept confidential (e.g., no one outside of the research team will see my survey.) I have read the consent document. I have asked all the questions that I have at this time. I voluntarily agree to be a participant in this study.	descriptive
8	[read_consent]	Please select one:	radio, Required<br>1 — I have read the consent document and I wish to participate in the study.<br>0 — I have read the consent document and I DO NOT wish to participate in the study.<br><br>Custom alignment: LV<br>Stop actions on 0
9	[f_name]<br><br>Show the field ONLY i f:<br>[read_consent] = '1'	First Name: [first_name]	descriptive, Required, Identifier
10	[l_name]<br><br>Show the field ONLY i f:<br>[read_consent] = '1'	Last Name: [last_name]	descriptive, Required, Identifier
11	[e_address]<br><br>Show the field ONLY i f:<br>[read_consent] = '1'	E-mail Address: [email]	descriptive, Required, Identifier
12	[consent_sign]<br><br>Show the field ONLY i f:<br>[read_consent] = '1'	By signing, you agree the above statements are true and have read and understood the consent document.	file (signature), Required, Identifier<br>Field Annotation: @stop

13	[date]	Today's Date:	text (date_mdy), Required<br>Field Annotation: @HIDEBUTTON @READONLY @TODAY
14	[irb_num]	Section Header: *The values below have been provided by the study team and do not need to be edited.*<br>IRB Number:	text, Required<br>Field Annotation: @DEFAULT = 'EH23797' @READONLY
15	[consent_version]	Consent Version:	text, Required<br>Field Annotation: @READONLY @DEFAULT='v3.0' @HIDEBUTTON
16	[version_date]	Consent Version Date:	text (date_mdy), Required<br>Field Annotation: @READONLY @DEFAULT='2023-12-08' @HIDEBUTTON
17	[consent_doc]	Please feel free to download a blank copy of the consent form for further review.	descriptive
18	[econsent_form_complete]	Section Header: *Form Status*<br>Complete?	dropdown<br>0 Incomplete<br>1 Unverified<br>2 Complete

Instrument: Usability And Acceptability Of Atrial Fibrillation (usability_and_acceptability_of_atrial_fibrillation) Enabled as survey

19	[healthcare_role]	Section Header: *Provider Information*<br>What is your role in healthcare?	radio<br>1. Physician (MD/DO/MBBS)<br>2. Advanced practice practitioner (nurse practitioner, physician assistant)<br>3. Other<br><br>Custom alignment: LV<br>Question number: 1
20	[other_healthcare_role]<br><br>Show the field ONLY if:<br>[healthcare_role] = '3.'	Define Other:	text<br>Custom alignment: LV
21	[years_of_practice]	How many years have you been in practice?	radio<br>1. < 1 Year<br>2. 1-5 Years<br>3. 6-10 Years<br>4. 11-15 Years<br>5. 16-20 Years<br>6. >20 Years<br><br>Custom alignment: LV<br>Question number: 2

22	[specialty]	What specialty of medicine do you practice?	radio<br>1. Family Medicine<br>2. Internal Medicine (Internal Medicine - Pediatrics)<br>3. Obstetrician/Gynecologist<br>4. Cardiology<br>5. Other<br><br>Custom alignment: LV<br>Question number: 3
23	[other_specialty]<br><br>Show the field ONLY if:<br>[specialty] = '5.'	Define Other:	text<br>Custom alignment: LV
24	[rural_urban]	Where is your primary clinical practice setting located?	radio<br>1. Rural/Small town (< 10,000 population)<br>2. Suburban/Urban (>10,000 population)<br><br>Custom alignment: LV<br>Question number: 4
25	[where_work]	Where do you primarily work? Select all that apply.	checkbox<br>1 where_work___1 Hospital based<br>2 where_work___2 Clinic based<br>3 where_work___3 Mix hospital/clinic<br>4 where_work___4 Skilled nursing facility, LTAC (Long Term Acute Care), Rehabilitation, or Assisted Living Facility<br>5 where_work___5 Other<br><br>Custom alignment: LV<br>Question number: 5
26	[other_where_work]<br><br>Show the field ONLY if:<br>[where_work(5)] = '1'	Define Other:	text<br>Custom alignment: LV
27	[freq_see_afib]	In an average 12-month time period, how many patients with atrial fibrillation (AFib) or atrial flutter do you typically see?	radio<br>1 I do not see atrial fibrillation or atrial flutter patients<br>2 1-9

			3 10-19<br>4 20-49<br>5 >50<br><br>Custom alignment: LV<br>Question number: 6<br>Stop actions on 1
28	[use_hm_tab]	Section Header: *A-Fib Care Gap Alert and Best Practice Advisory Alert*<br><br>Do you routinely use the Health Maintenance tab in Epic?	radio<br>1. Yes<br>2. No<br>3. I Don't Know<br><br>Custom alignment: LV<br>Question number: 7
29	[cg_intro_image]	Have you ever seen the "AFib/Aflutter without Anticoagulant" Care Gap alert (Figure 1)?	descriptive<br>Question number: 8
30	[cg_seen]		radio<br>1. Yes<br>2. No<br>3. I Don't Know<br><br>Custom alignment: LV
31	[cg_freq_used]	In the last 6 months, how often have you interacted with the "AFib/Aflutter without Anticoagulant" Care Gap alert?	radio, Required<br>1. Daily<br>2. Weekly<br>3. Monthly<br>4. Less than once a month<br>5. Never<br><br>Custom alignment: LV<br>Question number: 9
32	[cg_parts_helpful]	What aspects of the "AFib/Aflutter without Anticoagulant" Care Gap alert do you or would you find most helpful? Check all that apply.	checkbox<br>1. cg_parts_helpful___1 Awareness the patient has Atrial fibrillation or Atrial flutter diagnosis<br>2. cg_parts_helpful___2 Awareness the patient meets high-risk criteria based on AHA/ACC/HRS guidelines of CHA2DS2-VASc

			score (>/=2 male, >/=3 female)<br>3. cg_parts_helpful___3 Awareness the patient is not on anticoagulation<br>4. cg_parts_helpful___4 "X" to Defer/Delay alert<br>5 cg_parts_helpful___5 Check mark link to order set<br>6. cg_parts_helpful___6 Other<br>7. cg_parts_helpful___7 None<br><br>Custom alignment: LV<br>Question number: 10
33	[cg_other_parts_helpful]<br><br>Show the field ONLY if:<br>[cg_parts_helpful(6.)] = '1'	What other aspects do you or would you find helpful about the "AFib/Aflutter without Anticoagulant" Care Gap alert?	notes<br>Custom alignment: LV
34	[cg_why_parts_unhelpful]<br><br>Show the field ONLY if:<br>[cg_parts_helpful(7.)] = '1'	Please describe why you selected none.	notes<br>Custom alignment: LH
35	[persist_risk_strat_change]<br><br>Show the field ONLY if:<br>[cg_freq_used] = '1.' or [cg_freq_used] = '2.' or [cg_freq_used] = '3.' or [cg_freq_used] = '4.'	The "AFib/Aflutter without Anticoagulant" Care Gap alert utilizes the CHA2DS2-VASc calculator. It alerts if the patient is high-risk (>/=2 male >/=3 female). Knowing this, would you be more likely or less likely to start anticoagulation when seeing the "AFib/Aflutter without Anticoagulant" Care Gap alert?	radio<br>1. Extremely likely<br>2. Likely<br>3. Neutral<br>4. Unlikely<br>5. Extremely unlikely<br><br>Custom alignment: LV<br>Question number: 11
36	[likely_start_anticoag_convo]	How likely or unlikely are you to have a conversation with a patient with atrial fibrillation or atrial flutter about starting anticoagulation as a result of the "AFib/Aflutter without Anticoagulant" Care Gap alert?	radio<br>1. Extremely likely<br>2. Likely<br>3. Neutral<br>4. Unlikely<br>5. Extremely unlikely

			Custom alignment: LV<br>Question number: 12
37	[cg_likely_start_anticoag]<br><br>Show the field ONLY if:<br>[cg_freq_used] = '1.' or<br>[cg_freq_used] = '2.' or<br>[cg_freq_used] = '3.' or<br>[cg_freq_used] = '4.'	How likely or unlikely are you to start a patient with atrial fibrillation or atrial flutter on anticoagulation when responding to the "AFib/Aflutter without Anticoagulant" Care Gap alert?	radio<br>1. Extremely likely<br>2. Likely<br>3. Neutral<br>4. Unlikely<br>5. Extremely unlikely<br><br>Custom alignment: LV<br>Question number: 13
38	[cg_freq_influence_decision]<br><br>Show the field ONLY if:<br>[cg_freq_used] = '1.' or<br>[cg_freq_used] = '2.' or<br>[cg_freq_used] = '3.' or<br>[cg_freq_used] = '4.'	How frequently does the use of the "AFib/Aflutter without Anticoagulant" Care Gap alert influence your decision-making process during clinical encounters when the care gap is displayed?	radio<br>1. Always<br>2 Very often<br>3 Sometimes<br>4 Rarely<br>5 Never<br><br>Custom alignment: LV<br>Question number: 14
39	[cg_intro_edu_image]	Section Header: *AFib/Flutter Anticoagulation Order Panel*<br>The "AFib/Aflutter without Anticoagulant" Care Gap alert (Figure 2) opens the AFib/Flutter Anticoagulation order panel (Figure 3). It is displayed for patients diagnosed with atrial fibrillation or atrial flutter without anticoagulation that are high risk according to AHA/ACC/HRS guidelines (CHA2DS2-VASc score of >/=2 male, >/=3 female).	descriptive
40	[os_parts_helpful]	What aspects of the "AFib/Aflutter without Anticoagulant" Care Gap order panel do you find most helpful?Check all that apply.	checkbox<br>1. os_parts_helpful___1 Anticoagulation Treatment Recommendatio<br>2. os_parts_helpful___2 Anticoagulation Dosing information<br>3. os_parts_helpful___3 Recommended baseline labs to order<br>4. os_parts_helpful___4 Referral to the anticoagulation clinic<br>5. os_parts_helpful___5 Other<br>6. os_parts_helpful___6 None

			Custom alignment: LV<br>Question number: 15
41	[os_other_parts_helpful]<br><br>Show the field ONLY if:<br>[os_parts_helpful(5.)] = '1'	What other aspects do you find helpful about the "AFib/Aflutter without Anticoagulant" Care Gap order panel?	notes<br>Custom alignment: LH
42	[os_why_parts_unhelpful]<br><br>Show the field ONLY if:<br>[os_parts_helpful(6.)] = '1'	Please describe why you selected none.	notes<br>Custom alignment: LH
43	[cg_confort_aff_attitude]<br><br>Show the field ONLY if:<br>[cg_freq_used] = '1.' or<br>[cg_freq_used] = '2.' or<br>[cg_freq_used] = '3.' or<br>[cg_freq_used] = '4.'	Section Header: *16. How much do you agree with each of the following statements regarding the "AFib/Aflutter without Anticoagulation" Care Gap alert (AFib Care Gap Alert)?*<br><br>I feel comfortable responding to the AFib Care Gap Alert.	radio (Matrix)<br>1. 1. Strongly Disagree<br>2. 2. Disagree<br>3. 3. Neutral<br>4. 4. Agree<br>5. 5. Strongly Agree
44	[cg_effort_burden]<br><br>Show the field ONLY if:<br>[cg_freq_used] = '1.' or<br>[cg_freq_used] = '2.' or<br>[cg_freq_used] = '3.' or<br>[cg_freq_used] = '4.'	The AFib Care Gap Alert requires minimal effort to respond to.	radio (Matrix)<br>1. 1. Strongly Disagree<br>2. 2. Disagree<br>3. 3. Neutral<br>4. 4. Agree<br>5. 5. Strongly Agree
45	[cg_confident_self_effica]<br><br>Show the field ONLY if:<br>[cg_freq_used] = '1.' or<br>[cg_freq_used] = '2.' or<br>[cg_freq_used] = '3.' or<br>[cg_freq_used] = '4.'	I feel confident responding to the AFib Care Gap Alert.	radio (Matrix)<br>1. 1. Strongly Disagree<br>2. 2. Disagree<br>3. 3. Neutral<br>4. 4. Agree<br>5. 5. Strongly Agree
46	[cg_trust]<br><br>Show the field ONLY if:<br>[cg_freq_used] = '1.' or<br>[cg_freq_used] = '2.' or<br>[cg_freq_used] = '3.' or<br>[cg_freq_used] = '4.'	I trust the AFib Care Gap Alert.	radio (Matrix)<br>1. 1. Strongly Disagree<br>2. 2. Disagree<br>3. 3. Neutral<br>4. 4. Agree<br>5. 5. Strongly Agree
47	[cg_interefere_priorities2]	The AFib Care Gap Alert does not interfere with my other priorities while interacting with my	radio (Matrix)<br>1. 1. Strongly Disagree

	Show the field ONLY if: [cg_freq_used] = '1.' or [cg_freq_used] = '2.' or [cg_freq_used] = '3.' or [cg_freq_used] = '4.'	patients.	2. 2. Disagree<br>3. 3. Neutral<br>4. 4. Agree<br>5. 5. Strongly Agree
48	[cg_ethical_conseq]<br><br>Show the field ONLY if: [cg_freq_used] = '1.' or [cg_freq_used] = '2.' or [cg_freq_used] = '3.' or [cg_freq_used] = '4.'	The AFib Care Gap Alert does not have negative ethical consequences with use.	radio (Matrix)<br>1. 1. Strongly Disagree<br>2. 2. Disagree<br>3. 3. Neutral<br>4. 4. Agree<br>5. 5. Strongly Agree
49	[cg_coherence_howeffective2]<br><br>Show the field ONLY if: [cg_freq_used] = '1.' or [cg_freq_used] = '2.' or [cg_freq_used] = '3.' or [cg_freq_used] = '4.'	It is clear to me how the AFib Care Gap Alert helps prevent life threatening ischemic stroke for patients with atrial fibrillation or flutter.	radio (Matrix)<br>1. 1. Strongly Disagree<br>2. 2. Disagree<br>3. 3. Neutral<br>4. 4. Agree<br>5. 5. Strongly Agree
50	[susq1]<br><br>Show the field ONLY if: [cg_freq_used] = '1.' or [cg_freq_used] = '2.' or [cg_freq_used] = '3.' or [cg_freq_used] = '4.'	Section Header: *17. How much do you agree with each of the following statements regarding the "AFib/Aflutter without Anticoagulation" Care Gap Alert?*<br><br>I think that I would like to use this system frequently.	radio (Matrix)<br>1. 1. Strongly Disagree<br>2. 2. Disagree<br>3. 3. Neutral<br>4. 4. Agree<br>5. 5. Strongly Agree
51	[susq2]<br><br>Show the field ONLY if: [cg_freq_used] = '1.' or [cg_freq_used] = '2.' or [cg_freq_used] = '3.' or [cg_freq_used] = '4.'	I found this system unnecessarily complex.	radio (Matrix)<br>1. 1. Strongly Disagree<br>2. 2. Disagree<br>3. 3. Neutral<br>4. 4. Agree<br>5. 5. Strongly Agree
52	[susq3]<br><br>Show the field ONLY if: [cg_freq_used] = '1.' or [cg_freq_used] = '2.' or [cg_freq_used] = '3.' or [cg_freq_used] = '4.'	I thought the system was easy to use.	radio (Matrix)<br>1. 1. Strongly Disagree<br>2. 2. Disagree<br>3. 3. Neutral<br>4. 4. Agree<br>5. 5. Strongly Agree
53	[susq4]<br><br>Show the field ONLY if:	I think that I would need the support of a technical person to be able to use this system.	radio (Matrix)<br>1. 1. Strongly Disagree<br>2. 2. Disagree

	[cg_freq_used] = '1.' or<br>[cg_freq_used] = '2.' or<br>[cg_freq_used] = '3.' or<br>[cg_freq_used] = '4.'		3. 3. Neutral<br>4. 4. Agree<br>5. 5. Strongly Agree
54	[susq5]<br>Show the field ONLY i f:<br>[cg_freq_used] = '1.' or<br>[cg_freq_used] = '2.' or<br>[cg_freq_used] = '3.' or<br>[cg_freq_used] = '4.'	I found the various functions in this system were well integrated.	radio (Matrix)<br>1. 1. Strongly Disagree<br>2. 2. Disagree<br>3. 3. Neutral<br>4. 4. Agree<br>5. 5. Strongly Agree
55	[susq6]<br>Show the field ONLY i f:<br>[cg_freq_used] = '1.' or<br>[cg_freq_used] = '2.' or<br>[cg_freq_used] = '3.' or<br>[cg_freq_used] = '4.'	I thought there was too much inconsistency in this system.	radio (Matrix)<br>1. 1. Strongly Disagree<br>2. 2. Disagree<br>3. 3. Neutral<br>4. 4. Agree<br>5. 5. Strongly Agree
56	[susq7]<br>Show the field ONLY i f:<br>[cg_freq_used] = '1.' or<br>[cg_freq_used] = '2.' or<br>[cg_freq_used] = '3.' or<br>[cg_freq_used] = '4.'	I would imagine that most people would learn to use this system very quickly.	radio (Matrix)<br>1. 1. Strongly Disagree<br>2. 2. Disagree<br>3. 3. Neutral<br>4. 4. Agree<br>5. 5. Strongly Agree
57	[susq8]<br>Show the field ONLY i f:<br>[cg_freq_used] = '1.' or<br>[cg_freq_used] = '2.' or<br>[cg_freq_used] = '3.' or<br>[cg_freq_used] = '4.'	I found this system very cumbersome to use.	radio (Matrix)<br>1. 1. Strongly Disagree<br>2. 2. Disagree<br>3. 3. Neutral<br>4. 4. Agree<br>5. 5. Strongly Agree
58	[susq9]<br>Show the field ONLY i f:<br>[cg_freq_used] = '1.' or<br>[cg_freq_used] = '2.' or<br>[cg_freq_used] = '3.' or<br>[cg_freq_used] = '4.'	I felt very confident using the system.	radio (Matrix)<br>1. 1. Strongly Disagree<br>2. 2. Disagree<br>3. 3. Neutral<br>4. 4. Agree<br>5. 5. Strongly Agree
59	[susq10]<br>Show the field ONLY i f:<br>[cg_freq_used] = '1.' or<br>[cg_freq_used] = '2.' or	I needed to learn a lot of things before I could get going with this system.	radio (Matrix)<br>1. 1. Strongly Disagree<br>2. 2. Disagree<br>3. 3. Neutral

	[cg_freq_used] = '3.' or [cg_freq_used] = '4.'		4. 4. Agree<br>5. 5. Strongly Agree
60	[bpa_image]	Section Header: *Best Practice Advisory and Future Use*<br>Have you ever seen the "Best Practice Advisory for patients with atrial fibrillation and not on an anticoagulant or LAA device" (Figure 4)?	descriptive<br>Question number: 18
61	[afib_bpa_seen]		radio<br>1. Yes<br>2. No<br>3. I Don't Know<br><br>Custom alignment: LV
62	[bpa_helpful]	What is or would be helpful about the "Best Practice Advisory for patients with Atrial Fibrillation and not on an Anticoagulant or LAA device"? Select all that apply.	checkbox<br>1. bpa_helpful___1 Awareness the patient has atrial fibrillation or atrial flutter diagnosis<br>2. bpa_helpful___2 Awareness the patient meets high-risk criteria based on AHA/ACC/HRS guidelines of CHA2DS2-VASc score (>/=2 male, >/=3 female)<br>3. bpa_helpful___3 Awareness your patient is not on anticoagulation<br>4. bpa_helpful___4 Link to open SmartSet: AFIB Anticoagulation & Shared Decision Making<br>5. bpa_helpful___5 Option to defer/delay alert<br>6. bpa_helpful___6 Link to add "contraindication to anticoagulation therapy" to problem list<br>7. bpa_helpful___7 Link to add "presence of WATCHMAN (left atrial appendage closure device)"

			to the problem list<br>8. bpa_helpful___8 Link to add "S/P left atrial appendage ligation" to problem list<br>9. bpa_helpful___9 Link to up-to-date article: "Atrial fibrillation: Anticoagulant therapy to prevent embolization"<br>10. bpa_helpful___10 Other<br>11. bpa_helpful___11 None<br><br>Custom alignment: LV<br>Question number: 19
63	[bpa_other_helpful]<br><br>Show the field ONLY if:<br>[bpa_helpful(10.)] = '1'	What other aspects do you or would you find helpful about the Afib Best Practice Advisory?	notes<br>Custom alignment: LV
64	[bpa_none_helpful]<br><br>Show the field ONLY if:<br>[bpa_helpful(11.)] = '1'	Please describe why you selected none.	notes<br>Custom alignment: LV
65	[cg_bpa_preference_used]	Which tool are you more likely to respond to in Epic?	radio<br>1. "AFib/Aflutter without Anticoagulant" Care Gap alert<br>2. "Best Practice Advisory for patients with atrial fibrillation and not on an anticoagulant or LAA device"<br>3. Either<br>4. Neither<br>5. I Don't Know<br><br>Custom alignment: LV<br>Question number: 20
66	[alert_prefered]<br><br>Show the field ONLY if:<br>[cg_bpa_preference_used] = '4.' or [cg_bpa_preference_used] = '5.'	How would you prefer to be alerted of patients with atrial fibrillation that are high-risk (CHA2DS2-VASc >/=2 male, >/=3 females) and not on anticoagulation?	notes<br>Custom alignment: LH

67	[cg_recommend_to_others]<br><br>Show the field ONLY if:<br>[cg_freq_used] = '1.' or<br>[cg_freq_used] = '2.' or<br>[cg_freq_used] = '3.' or<br>[cg_freq_used] = '4.'	How likely is it that you would recommend the "AFib/Aflutter without Anticoagulant" Care Gap alert or "Best Practice Advisory for patients with atrial fibrillation and not on an anticoagulant or LAA device" to colleagues (or clinics) that are considering getting these tools?	radio<br>1. Extremely likely<br>2. Likely<br>3. Neutral<br>4. Unlikely<br>5. Extremely unlikely<br><br>Custom alignment: LV<br>Question number: 21
68	[ncg_freq_future_use]	The "AFib/Aflutter without Anticoagulant" Care Gap alert (Figure 2) utilizes the CHA2DS2-VASc calculator. It is displayed for patients diagnosed with nonvalvular atrial fibrillation or atrial flutter without anticoagulation that are high-risk according to (CHA2DS2-VASc score of >/=2 male, >/=3 female). In your future practice, how likely are you to respond to the "AFib/Aflutter without Anticoagulant" Care Gap alert or "Best Practice Advisory for patients with Atrial Fibrillation and not on an Anticoagulant"?	radio<br>1. Extremely likely<br>2. Likely<br>3. Neutral<br>4. Unlikely<br>5. Extremely unlikely<br><br>Custom alignment: LV<br>Question number: 22
69	[ncg_future_change_rec]	What should be changed to improve the "AFib/Aflutter without Anticoagulant" Care Gap alert or "Best Practice Advisory for patients with Atrial Fibrillation and not on an Anticoagulant or LAA device" tools? Please describe.	notes<br>Custom alignment: LH<br>Question number: 23
70	[number_watchmen]	In the past 12 months, how many patients with atrial fibrillation have you referred for consideration of WATCHMAN (left atrial appendage occlusion device)?	radio<br>1 I have never heard of a WATCHMAN device.<br>2 0<br>3 1-10<br>4 >10<br><br>Custom alignment: LV<br>Question number: 24
71	[sugg_close_gap]	Within Essentia Health, there are approximately 3,900 patients with atrial fibrillation who are not on anticoagulation and are at high-risk of ischemic stroke.How would you address this gap?	notes<br>Custom alignment: LV<br>Question number: 25
72	[transition_close]	Just hit submit! You are done! This is the end of our survey.We appreciate your participation in our study.Thank you for your time!	descriptive
73	[usability_and_acceptability_of_atrial_fibrillation_complete]	Section Header: *Form Status*<br><br>Complete?	dropdown<br>0 Incomplete<br>1 Unverified<br>2 Complete
